# 我如何开展CD19 CAR-T细胞治疗难治复发B细胞淋巴瘤患者的全程管理

**DOI:** 10.3760/cma.j.issn.0253-2727.2022.03.003

**Published:** 2022-03

**Authors:** 文斌 钱, 辉 刘

**Affiliations:** 浙江大学医学院附属第二医院血液内科，杭州 310009 Department of Hematology, the Second Affiliated Hospital, College of Medicine, Zhejiang University, Hangzhou 310009, China

嵌合抗原受体T细胞（CAR-T细胞）疗法是治疗淋巴瘤、白血病和多发性骨髓瘤等血液肿瘤重要的新方法。目前已有多款CAR-T细胞产品在美国、中国等多个国家获批上市，用于治疗难治、复发B细胞淋巴瘤[1]–[4]。与传统化疗、靶向药等疗法不同，CAR-T细胞疗法具有独特的毒副作用，包括细胞因子释放综合征（CRS）和免疫效应细胞相关神经毒性综合征（ICANS）等。作为新型“活”的细胞药物，在CAR-T细胞治疗之前如何评估入选患者、是否需要桥接细胞治疗；在治疗期间如何管理包括CRS等在内的早期不良反应；治疗后如何规范随访并管理中、远期不良反应以及CAR-T细胞治疗后再次复发、难治的患者如何处理等问题有待进一步厘清。CAR-T细胞治疗的全程管理对提高疗效和安全性至关重要。本文以一例复发、难治弥漫大B细胞淋巴瘤（DLBCL）患者接受CAR-T细胞治疗的整个过程为例，阐述和讨论笔者如何开展CD19 CAR-T细胞治疗难治复发B细胞淋巴瘤患者的全程管理。

一、典型病例

患者，男，63岁，2016年5月出现颈部双侧多发淋巴结肿大，病理活检诊断为EB病毒（EBV）阳性DLBCL，非特指型。免疫组化显示：CD19（+）、CD20（+）、CD30（+）、CD5（−）、CD10（−）、BCL2（+）、BCL6（−）、MUM1（+）、C-MYC（−）、ALK（−）和Ki-67（80％）；EBER阳性，血清EBV-DNA 5.44×10^6^拷贝/ml。患者合并慢性乙型病毒性肝炎，恩替卡韦治疗下HBV-DNA持续阴性。2个疗程R-CHOP、1个疗程R-GDP、3个疗程R2-MINE和1个疗程R2-DHAP后疗效评估为疾病进展。于2016年12月签署知情同意书后入组以4-1BB为共刺激域的CD19 CAR-T细胞临床试验。应用细胞分离机采集自体外周血单个核细胞（PBMC）制备CAR-T细胞。FC方案清除淋巴细胞后输注总量为1.26×10^8^个自体CAR-T细胞（2×10^6^/kg）。回输后第1天患者即出现发热，持续5 d，最高为38.7 °C，期间未出现低血压或低氧血症，按照当时最为常用的Lee等[5]制订的CRS分级标准为1级。经补液、退热等对症支持治疗和经验性抗感染治疗好转。回输后第12天患者中性粒细胞恢复出院。根据目前最常用美国移植和细胞治疗学会（ASTCT）指南[6]，判断为CRS 1级。CAR-T细胞回输第3个月，PET-CT提示患者获得完全代谢学缓解（CMR），且血清中未检测到EBV-DNA。

CAR-T细胞治疗4个月，患者出现低氧血症，未吸氧下血氧饱和度85％左右，无发热，肺部CT提示间质性肺炎和少量胸腔积液，支气管镜肺泡灌洗液未见异常，予免疫球蛋白10 g/d静脉注射，持续2周后，肺部CT提示间质性肺炎逐渐吸收改善。

患者后续定期门诊随访，并坚持口服恩替卡韦预防HBV激活。但于首次CAR-T细胞治疗后2年半再次出现下颌淋巴结肿大，PET-CT及淋巴结活检证实淋巴瘤复发，血清EBV-DNA 5.92×10^3^拷贝/ml，HBV-DNA始终低于检测下限。经评估，该患者入组靶向CD19、携带PD-1/CD28嵌合转换受体和以4-1BB为共刺激分子的新型CAR-T（CD19-PD-1/CD28-CAR-T）细胞挽救性治疗临床试验，FC方案预处理后，回输2×10^6^/kg CD19-PD-1/CD28-CAR-T细胞。CAR-T细胞回输后，患者发生ASTCT 2级CRS（发热合并低血压、低氧血症）和3级ICANS（短暂意识不清及抽搐），按照ASTCT指南的处理意见给予补液、抗癫痫、地塞米松及托珠单抗治疗后好转。治疗后3个月时PET-CT评估提示再次获得CMR。

然而在后续随访中，患者出现血细胞减少时间延长，贫血、粒细胞缺乏及血小板减少均达到3～4级血液学毒性，同时出现严重的B细胞缺陷，免疫球蛋白显著低下，两次骨髓穿刺检查均提示造血组织增生低下，定期接受输血、补充丙种球蛋白及升血细胞因子等对症支持治疗。但该患者在2020年12月又出现高热和低氧血症，偶有咯血，联系呼吸科会诊，给予支气管镜肺泡灌洗，明确为肺毛霉菌感染，住院接受两性霉素B脂质体、泊沙康唑等抗感染治疗，目前好转出院。尽管出现感染、血细胞减少持续时间延长等诸多不良反应，患者目前仍处于持续完全缓解的状态。

二、治疗前筛查和评估

1. 患者筛查及选择：商业化CD19 CAR-T细胞已经获批上市。国内也有以CD22、CD20、CD79b等为靶点的CAR-T细胞正在不同阶段研究进程之中。FDA批准的CD19 CAR-T细胞适应证为DLBCL、套细胞淋巴瘤（MCL）和转化的滤泡性淋巴瘤（TFL）。阿基仑赛注射液（axi-cel）和瑞基奥仑赛注射液（relma-cel）在国内获批上市，二线以上治疗失败的各种大B细胞淋巴瘤均是适应证。国际上ZUMA系列临床试验正在对慢性淋巴细胞白血病、其他小B细胞淋巴瘤等病种进行研究，并对CAR-T细胞治疗端口前移、联合治疗等进行有益的探索。

已有的众多真实世界研究数据显示，即使患者不符合ZUMA-1临床试验入排标准，经CD19 CAR-T细胞治疗也有机会获得持续缓解[7]。因此可根据临床实践和患者需求适度拓展接受CAR-T细胞治疗的人群。但为了最大程度保证安全，我们中心参考欧洲血液和骨髓移植学会（EBMT）、EBMT 和ISCT联合认证委员会（JACIE）、欧洲血液学协会（EHA）的临床最佳实践建议[8]，结合本中心经验，建议筛选CAR-T细胞治疗患者时要考虑以下标准：

（1）年龄：不设限制，更关注体能、合并疾病等情况，以及细胞单采术的年龄限制。

（2）体能评分：真实世界研究数据显示美国东部肿瘤协作组（ECOG）体力评分大于1分的患者，无进展生存（PFS）和总体生存（OS）时间均显著缩短。建议患者ECOG评分小于2分，或经过桥接治疗改善体能等总体状况再行CAR-T细胞治疗。

（3）预期寿命：建议大于6周，充分考虑治疗获益。

（4）肿瘤负荷：高肿瘤负荷特别是大肿块与CAR-T细胞治疗无效、重症CRS密切相关，建议经过适当桥接治疗改善后再行CAR-T细胞治疗。

（5）其他肿瘤：活动性肿瘤需要治疗的患者，建议暂缓CAR-T细胞治疗，有既往肿瘤病史患者充分告知风险后实施治疗。

（6）造血干细胞移植：既往接受自体造血干细胞移植患者可以接受CAR-T细胞治疗，且本中心前期研究数据显示移植并不影响CAR-T细胞疗效。异基因造血干细胞移植患者需停用免疫抑制剂至少1个月且无移植物抗宿主病（GVHD）发生。供者淋巴细胞回输建议至少停用4周并确保无GVHD发生。

（7）既往靶向CD19治疗病史：有研究表明CD19表达水平降低并不影响CD19 CAR-T细胞疗效，然而既往接受贝林妥欧单抗可能影响CD19 CAR-T细胞疗效，需慎重选择。国际研究表明，既往接受CD19 CAR-T细胞后再次进展患者，接受第二次CD19 CAR-T细胞的完全缓解率为20％，与常规挽救治疗并无明显差别，建议谨慎选择。本中心采用新型的具有PD-1/CD28嵌合转换受体的CD19 CAR-T细胞挽救性治疗6例患者，4例有效[9]。如有适宜临床研究，也可选择参加其他靶点如CD20、CD22或双靶点CAR-T细胞临床试验。

（8）免疫抑制治疗：影响T细胞功能，为相对禁忌证。但允许患者吸入性激素治疗或局部激素治疗。

（9）感染：活动性细菌或真菌感染为CAR-T细胞禁忌证，建议充分控制后再行细胞采集；某些活动性病毒感染为相对禁忌证，如带状疱疹病毒、单纯疱疹病毒等，可根据临床急迫程度充分权衡后进行CAR-T细胞治疗。而合并低水平HBV-DNA滴度的活动性乙肝患者，在充分接受抗HBV治疗的基础上，可以慎重选择CAR-T细胞治疗。乙肝或丙肝携带者在有效预防治疗的基础上可以接受CAR-T细胞治疗。

（10）中枢神经系统受累：中枢神经系统受累为相对禁忌证，众多临床试验包括ZUMA系列、本中心临床研究均将中枢神经系统受累作为排除标准。但TRANSCEND NHL 001研究则将继发中枢神经系统淋巴瘤患者纳入liso-cel治疗，且真实世界研究数据显示继发中枢神经系统受累患者接受CAR-T细胞治疗的疗效未见降低，安全性可控[10]。如患者接受CAR-T细胞治疗需要充分知情。

（11）造血功能：0.2×10^9^/L的CD3阳性淋巴细胞是CAR-T细胞治疗的最低阈值，但外周血CD3阳性细胞过低有可能体外扩增失败，对这类患者需要充分知情，或者推迟CAR-T细胞治疗，待CD3阳性细胞有所升高。另外，为保证采集顺利，建议血红蛋白大于80 g/L及血细胞比容大于0.24。此外，建议具有充分的骨髓造血储备功能以降低远期血细胞减少的风险。

（12）心功能：建议接受治疗的患者左室射血分数大于40％；心律失常患者在心内科协作下可权衡利弊接受CAR-T细胞治疗；少量心包积液未导致血流动力学不稳可接受CAR-T细胞治疗。

（13）肝肾功能：建议胆红素小于34 µmol/L，ALT、AST小于5倍上限值，肾小球肌酐清除率大于30 ml/min。由于肿瘤累及或压迫导致肝肾功能异常者，CAR-T细胞治疗谨慎考虑。

（14）生殖：充分告知并做好避孕措施，育龄期妇女拟接受治疗及CAR-T细胞回输前8天进行妊娠试验确保阴性。

2. PBMC采集的准备和注意事项：拟接受CAR-T细胞治疗的患者，在单采血细胞前2周避免使用淋巴细胞毒性药物，包括各种化疗药物及糖皮质激素。有少数研究表明伊布替尼可以降低PD-1表达并抑制2型辅助性T细胞（Th2）反应而提升CAR-T细胞疗效，可考虑细胞分离之前应用[11]。

3. 桥接治疗方案的选择和注意事项：桥接治疗指在淋巴细胞采集和最终输注CAR-T细胞之间采取治疗措施，延缓甚至逆转淋巴瘤进展，降低肿瘤负荷。目前众多临床试验允许入组患者接受桥接治疗[2]–[4],[12]。但需要注意尽量减少对后续清除淋巴细胞化疗及CAR-T细胞的不良影响。故患者情况允许，按本中心经验，ECOG评分小于3分、疼痛评分小于4分，非大包块、未产生临近脏器压迫症状的患者，无需接受桥接治疗。

比较常用的桥接治疗方案包括新型靶向药物，如来那度胺、BTK抑制剂和BCL2抑制剂等；根据患者情况也可采取受累野放疗、单抗类药物联合小剂量短疗程细胞毒药物或短疗程糖皮质激素治疗。

新近研究表明，某些桥接治疗有助于提升CAR-T细胞抗肿瘤效应，如前文提及的伊布替尼。BCL2抑制剂作用于肿瘤细胞，使其更易受死亡受体信号诱导凋亡，故能提升CAR-T细胞疗效。但考虑到BCL2通过抑制活化诱导的细胞死亡从而保护T细胞的效应，故目前建议应用到CAR-T细胞回输前停药[13]。

三、CAR-T细胞治疗时的管理

1. 预处理淋巴细胞清除（清淋）化疗：当收到CAR-T细胞制备成功并准备出库的通知时，需进行预处理清淋治疗，以期通过清除患者体内尚存淋巴细胞、消除免疫抑制因素、诱导共刺激分子等机制，减少患者免疫系统对CAR-T细胞的攻击，为其在体内扩增和存活创造有利条件。

本中心经验是在这个时间节点再次充分评估患者脏器功能、ECOG评分、感染状态等，确保患者对清淋化疗和后续CAR-T细胞回输的安全性和可耐受性。

为了避免清淋对CAR-T细胞的毒性，停止清淋化疗到CAR-T细胞回输至少间隔48 h以上。个别情况下CAR-T细胞回输延迟，与清淋化疗间隔3周以上，则根据患者骨髓抑制情况，再次给予FC方案预处理化疗，并适当调整化疗的剂量和疗程。

清淋方案建议采用ZUMA-1研究给予的FC方案，即氟达拉滨30 mg·m^−2^·d^−1^，−5～−3 d；环磷酰胺500 mg·m^−2^·d^−1^，−5～−3 d。对年龄大、血象偏低或体能状态差的患者，我们也采用调整剂量的方案，即氟达拉滨25 mg·m^−2^·d^−1^，−5～−3 d；环磷酰胺900 mg/m^2^，−5 d。

2. 细胞回输的注意事项：即使relma-cel仅需数毫升液体静脉缓慢推注治疗，但考虑到后续液体管理，建议接受CAR-T细胞治疗患者需放置中心静脉置管，常用经外周静脉置入中心静脉导管（PICC）、中心静脉置管（CVC）和输液港三种置管模式。

CAR-T细胞回输时注意密切监测患者各项生命体征，避免使用去白细胞输血滤器，一般不做任何预防输液反应的干预，如抗组胺类药物或糖皮质激素。

回输后密切注意CRS、ICANS的识别，注意肿瘤溶解综合征的监测，即需要密切监测细胞因子、血常规、肝肾功能、电解质、凝血功能及D-二聚体、炎症因子指标C反应蛋白、铁蛋白、CAR-T细胞拷贝数以及免疫球蛋白。特别要关注早期发生的发热等CRS临床表现。根据国际相关研究和我们的经验，早期出现CRS往往是严重的CRS。

另外需注意病房备药，如托珠单抗、左乙拉西坦、血管活性药物、糖皮质激素（包括地塞米松针剂和甲泼尼龙针剂），以备及时有效干预。

四、CAR-T细胞治疗后的管理

1. CRS及ICANS的监测和处理：关于不良反应分级和处置国内外有多项标准。早期本中心采用Lee等[5]标准。目前国际上多推荐应用ASTCT标准[6]。不同标准的CRS和ICANS分级会有明显的差异。应用ASTCT标准进行分级明显偏低。关于CRS及ICANS的分级和处理方法，建议采用《嵌合抗原受体T细胞治疗相关神经系统毒副反应管理中国专家共识（2022年版）》的推荐[14]。

在加强临床监测的同时，也需积极注意鉴别诊断，如发热是否合并感染，癫痫是否合并脑血管意外等。需要重视的是，可能会出现浆膜腔积液、脏器功能不全等未列入ASTCT的不良反应，往往伴随CRS或ICANS出现；一些患者的胸腹水检测可以测到CAR阳性细胞，因此也需给予激素或托珠单抗处理。此外，还观察到某些患者虽然未出现血压下降，但已经合并尿量减少、出入量不平衡的情况，提示可能存在肾脏有效灌注不足，需要在液体管理的基础上尽快启动激素或托珠单抗治疗。

严重CRS（sCRS）的治疗[15]：国际文献定义sCRS为体温≥38 °C至少持续3 d、2种以上外周血细胞因子倍增≥75倍或1种细胞因子倍增≥250倍、需要升压药治疗的低血压、低氧血症、神经系统改变。对出现sCRS或3～4级CRS的患者应密切监测生命体征及神志变化。这类患者需每日多次评估和综合考虑重要脏器功能，及时处理。此外，多学科协作诊疗在sCRS治疗过程中必不可少。

2. 其他常见不良反应和对症支持治疗：

（1）感染：清除淋巴细胞化疗所引发的粒细胞缺乏、患者B淋巴细胞缺如和CRS等因素与感染高发有关。有研究报道，CD19 CAR-T细胞回输28 d内有18％患者合并感染，包括细菌、真菌及病毒[16]。感染患者出现发热，与CRS难以鉴别。需要反复多次病原体培养，结合细胞因子水平、降钙素原、G试验及GM试验，并引入感染二代测序技术，积极鉴别诊断。当难以鉴别诊断时，按照《中国中性粒细胞缺乏伴发热患者抗菌药物临床应用指南（2020年版）》给予经验性抗感染治疗[17]。

我们中心前期数据总结发现预防性抗病毒治疗有助于降低CD19 CAR-T细胞治疗后短期和远期带状疱疹发生率[18]。同时按照NCCN指南和临床实践，建议预防性阿昔洛韦抗病毒治疗减少带状疱疹发生。对于CAR-T细胞治疗期间出现Ⅲ～Ⅳ度骨髓抑制或中性粒细胞减少伴发热患者推荐预防性抗真菌治疗[14]。

（2）假性进展：我国多个中心有患者出现局部CRS，即在CAR-T细胞回输后，肿块由于局部炎症反应，有可能在短期之内出现进展，甚至引发压迫症状[19]。如有条件穿刺并明确病灶性质，则有助于临床判断，需要密切监测，必要时应用糖皮质激素进行管理，没有明确疾病进展不进行挽救性化疗。

3. 升血细胞药物的应用及注意事项：单核巨噬细胞活化是CRS发生、发展的重要原因，因此应避免使用粒-巨噬细胞集落刺激因子（GM-CSF）。而粒细胞集落刺激因子（G-CSF）能否应用，目前国际上也存在争议[20]–[21]。本中心目前对于重度粒细胞缺乏（中性粒细胞绝对计数<0.2×10^9^/L）患者或合并严重感染患者，积极给予G-CSF治疗。

对于血小板减少患者，我们中心尝试rhTPO或口服TPO激动剂，均有助于缩短患者血小板低下时间，减少血小板输注。

由于FC方案中氟达拉滨的应用，为避免输血相关移植物抗宿主病（TA-GVHD）的发生，针对CAR-T细胞回输后3个月内急需输血的患者，建议采取去白细胞滤器输血策略。

4. 噬血细胞性淋巴组织细胞增生症/巨噬细胞活化综合征：相对罕见，常发生于CRS恢复期和伴发CRS过程中[14]。

五、远期随访和管理

1. 二次肿瘤：本中心自2017年底开始CAR-T细胞临床研究，目前为止没有发现二次肿瘤发生率的显著升高。而真实世界随访数据显示axi-cel回输后有3％发生二次肿瘤，骨髓增生异常综合征（MDS）常见。因此，在随访过程中仍需引起高度警惕。

2. 血细胞减少时间延长的管理：一部分患者接受CAR-T细胞回输后，会发生长时间的一系或多系血细胞减少。对于这部分患者建议定期行骨髓穿刺检查，排查MDS或其他继发的血液系统疾病；对于合并感染或出血的患者，及时给予造血生长因子，并适时给予血制品输注。

3. B细胞功能缺陷的管理：尽管B细胞功能缺陷在CD19 CAR-T细胞回输后持续相当长时间，并导致患者感染风险增加，但大部分患者并未出现显著感染或危及生命。因此，本中心仅对活动性感染的患者采取丙种球蛋白替代治疗。

B细胞功能缺乏也可能导致HBV再激活，甚至引发肝功能衰竭[22]。文献报道，20％合并慢性HBV感染的淋巴瘤患者接受CD19 CAR-T细胞引发HBV再激活，提示CAR-T细胞输注后定期随访乙肝三系的重要性，而积极调整抗乙肝治疗能有效控制乙肝病毒拷贝数并防止重症肝炎的发生[23]。

4. CAR-T细胞治疗后疾病复发或难治的管理：目前仍有约半数患者在CAR-T细胞治疗缓解后疾病复发或者难治。对于此类患者，国际上采用常规治疗，包括放疗、化疗、PD-1单抗和来那度胺为基础的联合治疗以及二次CD19 CAR-T细胞治疗。一些新的靶向药物如BCL-2抑制剂、西达本胺等可以探索研究。本中心小样本研究提示新型第四代CAR-T细胞进行挽救性治疗可能是一种选择。新靶点和双靶点是未来的方向。

五、结语

作为一种全新的抗肿瘤治疗方法，CAR-T细胞为B细胞淋巴瘤患者带来了新的希望。但我们对CAR-T细胞疗法的认知有待更多的临床探索。全程管理对提高CAR-T细胞疗法的疗效和安全性非常重要，值得我们进一步关注和实践。
